# Optimization of Extraction Parameters for Enhanced Recovery of Bioactive Compounds from Quince Peels Using Response Surface Methodology

**DOI:** 10.3390/foods12112099

**Published:** 2023-05-23

**Authors:** Vassilis Athanasiadis, Theodoros Chatzimitakos, Eleni Bozinou, Konstantina Kotsou, Dimitrios Palaiogiannis, Stavros I. Lalas

**Affiliations:** Department of Food Science & Nutrition, University of Thessaly, Terma N. Temponera Str., 43100 Karditsa, Greece; vaathanasiadis@uth.gr (V.A.); tchatzimitakos@uth.gr (T.C.); empozinou@uth.gr (E.B.); kkotsou@agr.uth.gr (K.K.); dipaleog@med.uth.gr (D.P.)

**Keywords:** quince peels, extraction, response surface methodology, ultrasound, pulsed electric field, polyphenols, ascorbic acid, principal component analysis, partial least squares analysis

## Abstract

Quinces are well known for their multiple health benefits, including antioxidant, hypoglycemic, antimicrobial, anti-inflammatory, anticarcinogenic, etc., properties. Despite the widespread utilization of various plant parts, the peel has been largely ignored in the industry. In this study, we explored the effects of different extraction parameters, such as temperature, time, and composition of the extraction solvent, and techniques such as ultrasound (US) and a pulsed electric field (PEF), either alone or in combination, and optimized these parameters using a response surface methodology (RSM) to enhance the extraction of bioactive compounds such as chlorogenic acid, total polyphenols, flavonoids, and ascorbic acid from waste quince peels. From our results, it was apparent that quince peels are a great source of many bioactive compounds with high antioxidant activity. More specifically, after principal component analysis (PCA) and partial least squares (PLS) analysis, quince peels contain high levels of total polyphenols (43.99 mg gallic acid equivalents/g dw), total flavonoids (3.86 mg rutin equivalents/g dw), chlorogenic acid (2.12 mg/g dw), and ascorbic acid (543.93 mg/100 g dw), as well as antioxidant activity of 627.73 μmol AAE/g and 699.61 μmol DPPH/g as evidenced by FRAP and DPPH assays, respectively. These results emphasize the potential of utilizing quince peels as an eco-friendly and cost-effective source of bioactive compounds with various applications in the food and pharmaceutical industries for the prepared extracts.

## 1. Introduction

The quince tree (*Cydonia oblonga* Mill.) is a fruit tree of the *Rosaceae* family that can live up to 50 years [1,2]. *C. oblonga* is widely cultivated in countries of the Mediterranean region, such as France, Spain, and Greece. It has a rich history since ancient Greece when it was used as a symbol of affection, fertility, and happiness, known as the “Golden Apple” [3,4]. To date, its cultivation has considerable financial value, with Turkey producing ~25% of the world’s production, followed by China, Iran, Argentina, and Morocco [5].

The quince tree and its fruits are among the most exploitable species since the flesh and leaves are used in the food industry, while the wooden parts are used in the woodworking industries. Quinces are fruits rich in many bioactive compounds that bestow antioxidant, hypoglycemic, antimicrobial, anti-inflammatory, anticarcinogenic, antiallergic, and anti-ulcerative properties [6]. Apart from being eaten raw, the flesh is soft and juicy [2] and is mainly used to make jelly, jam, and compote [7,8,9]. Similarly, the leaves are considered a valuable source of bioactive compounds and a rich source of phenolic compounds, even richer than the fruit [10,11,12,13,14]. To this end, quince leaves are consumed as a beverage since they have been proven to cure diarrhea, heart palpitations, and various eye diseases [14,15]. Finally, the wooden parts of the quince tree are exceptionally durable, resistant to rot, and suitable for detailed woodworking, making it a popular material for furniture production [2].

On the basis of the above, the peel of the quince is the only part of the plant that is not valorized and discarded. Food waste is one of the major worldwide concerns as it leads to multiple economic and environmental problems. This is easily understood by the fact that 23% of the ecological footprint of each person is attributable to the food they buy and their waste [16,17,18,19]. Climate change is associated with the food waste that is produced, due to the energy and water consumed for the production of the food, resulting in excessive greenhouse gas production and consumption of unsustainable water resources. For instance, in the USA, the food wasted each year required a quarter of the country’s freshwater for its production [16,18,20]. As a result, efforts are being made to raise awareness of the issue and encourage a change in attitudes toward food waste [21]. To this end, there is a pressing need to find ways to reduce waste and promote the utilization of all parts of plants. In this context, the valorization of quince peels can assist in the achievement of the above aim. By optimizing the extraction of bioactive compounds from quince peels, waste can be reduced, and at the same time, extracts are being prepared that can be further utilized in various industries, including the food and pharmaceutical industries. This can not only contribute to more sustainable use of the available resources but also lead to the development of new products with potential health benefits. Therefore, it is crucial to explore ways to optimize the extraction of bioactive compounds from quince peels and promote their valorization.

The aim of this study is to evaluate the potential of quince peels as a source of bioactive compounds such as chlorogenic acid, polyphenols, flavonoids, and ascorbic acid and to optimize the extraction process of these compounds to reduce food waste. The effects of different extraction parameters, such as temperature, time, and solvent composition, were studied. Extraction was carried out using a classical stirring approach. However, the use of ultrasound and a pulsed electric field, either alone or in combination, was further exploited as a pretreatment step to further maximize the extraction yield of the bioactive compounds.

## 2. Materials and Methods

### 2.1. Chemicals and Reagents

All information regarding the chemicals and reagents used is given in the Supplementary Materials.

### 2.2. Sample Preparations and Extraction Process

Quinces (*Cydonia oblonga*) were supplied in October 2022 by local farmers in Pella county (Central Macedonia, Greece). Randomly, 5 trees and 8 fruits from each tree were chosen. Additionally, the direction in which the fruit faces the sun was examined. The quinces were carefully washed with tap water and dried using a paper towel. The peel was removed manually with a knife (with the least amount of peeling loss), cut into smaller pieces, and placed in a Biobase BK-FD10P (Shandong, China) lyophilizer to remove the water. The lyophilized peels were powdered and placed in sieves to separate them according to size.

For the extractions, 1 g of the powdered quince peels (average particle diameter of 106 μm) was placed in a glass beaker along with 20 mL of the extraction solvent. The composition of the extraction solvent (ethanol used in different concentrations, 0–100% *v*/*v*) is given in Table 1. Extraction was carried out by stirring (ST) at 500 rpm at different temperatures and variable times, according to Table 1. Ultrasound (US) and pulsed electric field (PEF) treatments were also applied to the samples, according to Table 1. For both US and PEF treatments, a hydration step was carried out on the dried material by leaving the dry powder submerged in the solvent for 10 min, following a 20 min treatment with either technique. Then, the samples were subjected to ST extraction, as mentioned above. For the combined treatment, a 10 min hydration step was performed followed by 20 min of PEF and 20 min of US, prior to ST. In all cases, after extraction, the sample was centrifuged for 10 min at 4500 rpm and the supernatant was retracted and stored at −40 °C until further analyzed. 

For PEF processing of the samples, two custom stainless steel chambers (Val-Electronic, Athens, Greece), a mode/arbitrary waveform generator (UPG100, ELV Elektronik AG, Leer, Germany), a digital oscilloscope (Rigol DS1052E, Beaverton, OR, USA), and a high-voltage power generator (Leybold, LDDi-dactic GmbH, Huerth, Germany) were used [22,23]. The electric field density was set to 1.0 kV/cm, the pulse period was 1 ms (frequency: 1000 Hz), and the pulse duration was 10 μs.

For US treatment, the temperature was maintained at 30 °C in the Elmasonic P machine (Elma Schmidbauer GmbH, Singen, Germany), operating at 37 kHz.

### 2.3. Response Surface Methodology (RSM) Optimization and Experiment Design

The total polyphenols (TPC), mainly chlorogenic acid, total flavonoids (TFC), and ascorbic acid were extracted with the highest possible yield using an RSM approach (the antioxidant activity was assessed using the FRAP and DPPH assays). Therefore, the design’s objective was to maximize the concentration of chlorogenic acid as well as the values of TPC, TFC, FRAP, DPPH, and ascorbic acid. This was accomplished by optimizing the extraction technique, the solvent (ethanol, EtOH) concentration (*C*, % *v*/*v*), the extraction time (*t*, min), and the extraction temperature (*T*, °C). The basis for the optimization was an experiment with a major effect screening design and 20 design points. The process variables were established in five levels in accordance with the experimental design. The coded and actual levels are listed in Table 1. The overall model significance (R^2^, *p*) and the significance of the model (equations) coefficients were assessed at a minimum level of 95% using the analysis of variance (ANOVA) and summary-of-fit tests.

A second-order polynomial model, shown in the following Equation (1), was also used to predict the response variable as a function of the examined independent factors:

(1) $$Y_{k}=\beta_{0}+\sum_{i=1}^{2}\beta_{i}X_{i}+\sum_{i=1}^{2}\beta_{\mathrm{ii}}X_{i}^{2}+\sum_{i=1}^{2}\sum_{j=i+1}^{3}\beta_{\mathrm{ij}}X_{i}X_{j}$$

where *Y_k_* is the predicted response variable; *X_i_* and *X_j_* are the independent variables; *β*_0_, *β_i_*, *β_ii_*, and *β_ij_* are the intercept, regression coefficients of the linear, quadratic, and interaction terms of the model, respectively.

The greatest peak area and the effect of a significant independent variable on the response were both determined using the RSM. The 3D surface response graphs were constructed to visually represent the model equation.

### 2.4. Determination of Total Polyphenol Content (TPC)

Determination of TPC was based on a previous procedure [24]. Information is given in the Supplementary Materials. 

### 2.5. Determination of Total Flavonoid Content (TFC)

Determination of TFC was based on a previous procedure [25]. Information is given in the Supplementary Materials.

### 2.6. Ferric Reducing Antioxidant Power (FRAP) Assay

Evaluation of FRAP assay was based on a previous procedure [26]. Information is given in the Supplementary Materials.

### 2.7. DPPH Radical Scavenging Activity

Evaluation of DPPH radical scavenging activity was based on a previous procedure [24]. Information is given in the Supplementary Materials.

### 2.8. Ascorbic Acid Content

A colorimetric test developed by Dani et al. [27] was used to measure the ascorbic acid concentration. To 900 μL of 10% *w*/*v* trichloroacetic acid, 100 μL of quince peel extract was added. The resultant solution was then mixed with 500 μL of 10% (*v*/*v*) Folin-Ciocalteu reagent. After 10 min, the absorbance at 760 nm was measured. Ascorbic acid (10–80 mg/L) was used to prepare a standard curve.

### 2.9. HPLC-Based Determination of the Chlorogenic Acid Content and Other Phenolic Compounds

The sample extracts were analyzed using an HPLC system [28]. Information is given in the Supplementary Materials. 

### 2.10. Statistical Analysis

The experimental design, as well as the statistical analysis related to the response surface methodology (RSM), the distribution analysis, the principal component analysis (PCA), and the partial least squares (PLS) analysis, was carried out using JMP^®^ Pro 16 software (SAS, Cary, NC, USA). The extraction procedures were carried out three times, and the quantitative analysis was performed in triplicate, resulting in a total of (3 × 3) measurements for each sample. The results are presented as average values with the corresponding standard deviations.

## 3. Results and Discussion

### 3.1. Extraction Optimization

**Figure 1:**
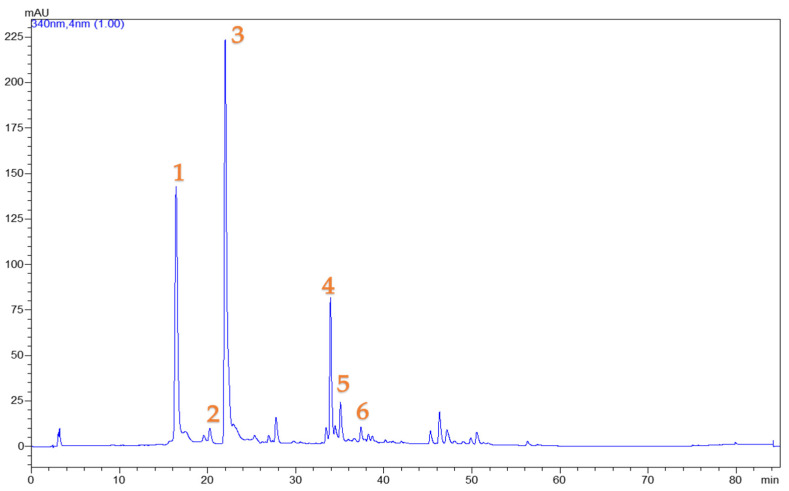
Exemplary HPLC chromatogram at 320 nm of a quince peel extract demonstrating the phenolic compounds that were identified. Peak 1: Chlorogenic acid; Peak 2: Neochlorogenic acid; Peak 3: Cryptochlorogenic acid; Peak 4: Rutin; Peak 5: Quercetin 3-*O*-galactoside; Peak 6: Kaempferol 3-*O*-β-rutinoside.

The valorization of food waste to isolate bioactive compounds is a constantly rising key research topic. Total polyphenols and ascorbic acid that can be found in significant quantities in quince peel can be of great value to the food industry [29]. To ensure maximum extraction yield of these bioactive compounds, various extraction parameters such as solvent composition (water, ethanol, and their mixtures 25, 50, and 75% (*v*/*v*), extraction time (ranging between 15 and 150 min), and extraction temperature (studied in the range 20–80 °C) were tested. In order for the extraction medium to be sufficient and yield optimal results, earlier research and preliminary experiments determined that the solid:solvent ratio should be 1:20 (i.e., 1 g of quince peel and 20 mL of solvent) [19]. In addition to the basic extraction (ST), two techniques characterized by low energy consumption that can be easily adopted for the preparation of extracts on a larger scale were tested either individually, before ST, as well as used one after the other to examine their combined effect (PEF was performed first followed by US). To assess the impact of each extraction factor and to enhance the compound extraction yield, a response surface methodology (RSM) was employed. Using ANOVA and summary-fit-tests to compare the measured values to the predicted ones, the efficacy of the response surface and model fit was evaluated. All measured responses for each prepared extract are presented in Table 2, including chlorogenic acid, which was the main polyphenol in the extraction. In addition, in Table 3, the concentrations of other polyphenolic compounds [neochlorogenic acid (0.54–1.51 mg/g dw), cryptochlorogenic acid (0.12–0.23 mg/g dw), rutin (0.43–0.81 mg/g dw), quercetin 3-*O*-galactoside (0.05–0.14 mg/g dw), and kaempferol 3-*O*-β-rutinoside (0.06–0.09 mg/g dw)] detected in the extracts are presented, while a representative chromatogram is given in Figure 1.

Neochlorogenic acid has also been identified in a previous study in the flesh and peel of quince [30]. The amount of this phenolic compound in the flesh can be characterized as ‘poor’ as it was found to be nearly 0.0796 mg/g. However, compared to the minimum amount found in the extracts prepared in our case, the amount of the same compound in the peel was determined to be 0.2916 mg/g, an increase of 85% [30]. Additionally, 0.51 ± 0.32 mg/g of cryptochlorogenic acid (4-*O*-caffeoylquinic acid) was also identified in the peel, in accordance with prior research [30]. Rutin is a phenolic compound that is recorded to exist in large amounts in the leaves of the quince tree (15.95 ± 0.03 mg/g). However, as reported previously, the amount in the peel is quite low [11,30]. Quercetin, another phenolic compound, is reported to exist in the plant’s flesh rather than the peel, at values of about 0.24 mg/g [31]. This was confirmed by our findings, where quercetin was detected in the peel at amounts that were lower than those found in the flesh as previously reported. Last but not least, kaempferol 3-*O*-β-rutinoside was only detected in quince peel and not in the pulp, according to a previous study [30]. Its amount was about 0.0369 mg/g, which is less than but comparable to the results of the present study.

Examination of the extraction parameters is of paramount importance, since it can minimize the use of resources (e.g., solvents, time, energy, etc.) and at the same time achieve maximum extraction efficiency. Therefore, it is important to optimize such parameters to render the whole process more environmentally friendly [19]. The composition of the solvent is one of the most important parameters since the extraction of the compounds is based on the properties of the solvent. More specifically, according to the polarity of the solvent, compounds are either extracted to a higher or a lower extent [23]. Polyphenols are compounds of medium polarity and as such, they cannot be effectively extracted by water. Therefore, the use of organic solvents such as ethanol is often employed to enhance the extraction performance [22,23,24,25]. This is in accordance with our results, since it was found that optimum extraction of polyphenols was achieved with higher percentages of ethanol in the extraction solvent. In addition, the combination of other green extraction techniques with the standard extraction technique was often proved to be more efficient [19,22,23,24,28]. This is because they cause further disruption of cellular membranes and therefore compounds are more easily extracted. This was previously showcased in other studies, where it was found that the use of PEF or US or even the combination of the two, prior to the main extraction step, proved to be more efficient [19,22,23,24,28].

**Figure 2:**
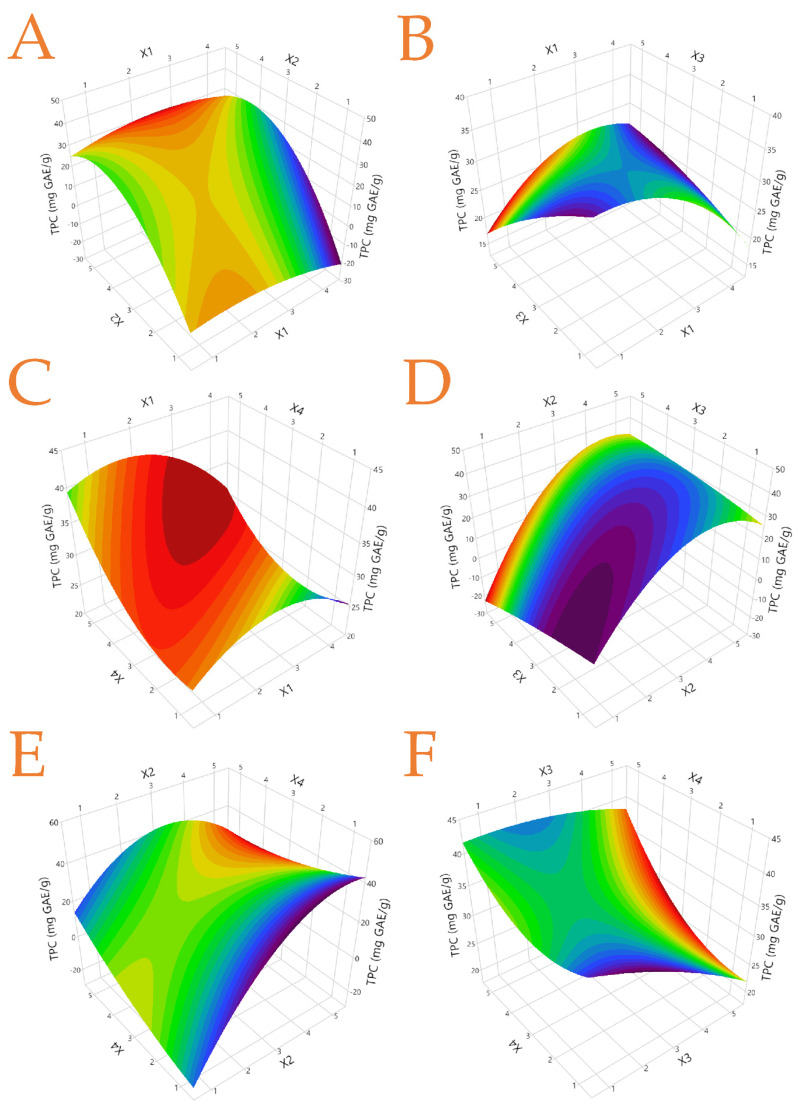
Three-dimensional graphs depicting the effect of the process variables considered in the response (Total Polyphenol Content-TPC, mg GAE/g) to optimize the extraction of the quince peel, using various extraction techniques and hydroethanolic solutions. Plot (**A**), covariation of *X*_1_ and *X*_2_; plot (**B**), covariation of *X*_1_ and *X*_3_; plot (**C**), covariation of *X*_1_ and *X*_4_; plot (**D**), covariation of *X*_2_ and *X*_3_; plot (**E**), covariation of *X*_2_ and *X*_4_; plot (**F**), covariation of *X*_3_ and *X*_4_.

**Figure 3:**
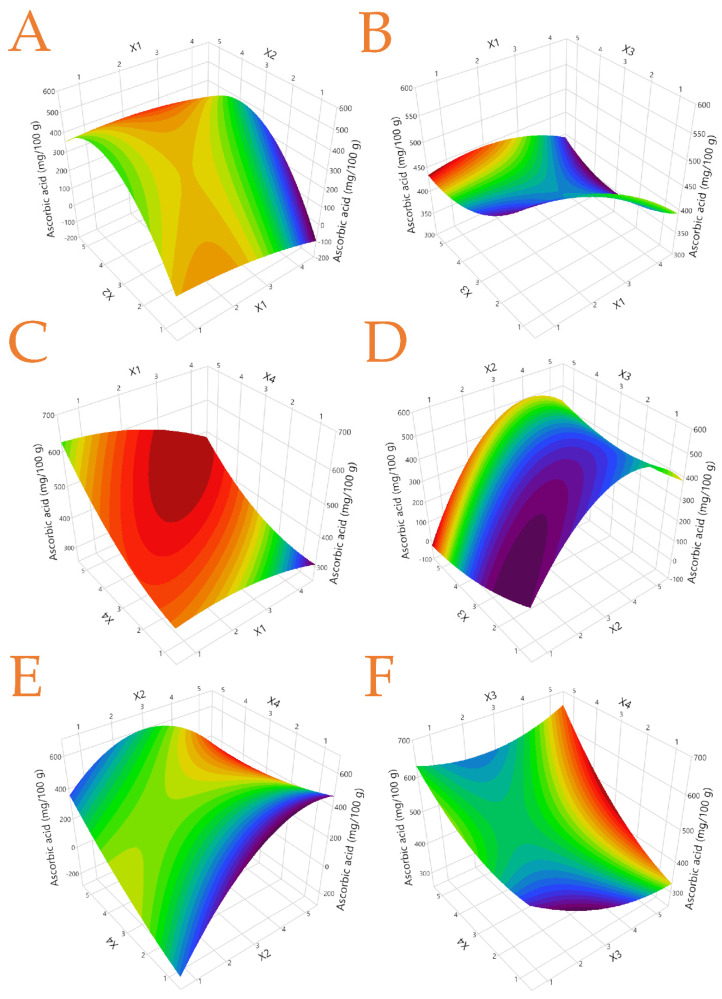
Three-dimensional graphs depicting the effect of the process variables considered in the response (Ascorbic acid, mg/100 g) to optimize the extraction of the quince peel, using various extraction techniques and hydroethanolic solutions. Plot (**A**), covariation of *X*_1_ and *X*_2_; plot (**B**), covariation of *X*_1_ and *X*_3_; plot (**C**), covariation of *X*_1_ and *X*_4_; plot (**D**), covariation of *X*_2_ and *X*_3_; plot (**E**), covariation of *X*_2_ and *X*_4_; plot (**F**), covariation of *X*_3_ and *X*_4_.

Finally, in Table 4, the statistical parameters, second-order polynomial equations (models), and coefficients (coefficients > 0.94) obtained for each model are presented, suggesting a good fit for the developed models. Plots of the actual response versus the predicted response for each examined parameter as well as the desirability functions are given in Figures S1–S6. Three-dimensional response plots for TPC and ascorbic acid are given in Figure 2 and Figure 3 while three-dimensional response plots for the rest of the responses are in Figures S7–S10.

### 3.2. Chlorogenic Acid Content of the Extracts

Chlorogenic acid is beneficial for human health as it prevents weight gain, insulin resistance, and the accumulation of fat in the liver [32,33] while it is also known for its anti-cancer properties [34,35,36]. Using an ethanolic solution (75%), US prior to ST, in a short time and at a relatively high temperature, the maximum amount of chlorogenic acid can be obtained from the quince peel. Specifically, the maximum amount that could be isolated was 2.30 ± 0.41 mg/100 g dw (Table 5). The amounts of chlorogenic acid have been extensively investigated in a related species of quince, apples [37,38,39,40]. In a study where the peel of three apple species, Royal Gala, Golden Delicious, and Fuji, was studied, ~0.12, ~0.06, and ~0.16 mg/g dw of chlorogenic acid were found [41,42]. 

### 3.3. Total Polyphenol Content (TPC) and Flavonoids Content (TFC) of the Extracts

According to the results (Table 2), it can be observed that depending on the technique used, as well as the various extraction parameters, the amount of polyphenols in the extract can vary from 6.50 mg GAE/g to up to 38.39 mg GAE/g. The use of ethanol and the long extraction time proved to be necessary for the extraction of the highest possible amount of polyphenols, whereas elevating the temperature was not found to further increase the TPC content and extraction at 35 °C was found to be adequate. A combination of US and ST was found to be the optimal extraction method. In an earlier study where methanolic extraction of the peel of quinces of the same species was performed, the amount of polyphenols detected was 6.3 mg GAE/g dw, the same as the lowest amount found in the present study [43]. This suggests that the optimization of the extraction conditions was far from necessary in order to extract the maximum amount of polyphenols. 

Flavonoids are a class of polyphenolic secondary metabolites found in plants [44]. However, little is known about the potential effects of sun radiation on fruits on the production of secondary metabolites in the peel. Henry-Kirk et al. [45] claim that even after the fruit has been stored, the action of UV light from sunshine continues to have an impact on the formation of secondary metabolites. Following a study conducted by Khan et al. [46], flavonoids identified in *Cydonia oblonga* Miller (quince) were found to be 0.779 ± 0.074 mg quercetin equivalents/g dw. According to our results, a maximum TFC of 3.49 ± 0.26 mg RtE/g dw (Table 5) can be achieved. As in the case of TPC, the combination of US + ST emerged as the optimal method of extraction, while the extraction time was very short, just 30 min, and the temperature was higher (65 °C). Once again, ethanol was found to be a suitable solvent at a ratio of 75% mixed with water.

### 3.4. Antioxidant Properties of the Extracts

The antioxidant capacity of the samples was assessed with two different assays (FRAP and DPPH). It turned out that ethanol played a crucial role in the extraction and complete isolation of antioxidants, as in both cases the optimal antioxidant activity was found in the extracts with 100% ethanol used as extraction solvent. The extraction time seemed to play an equally significant role, as the longer the extraction time, the higher the antioxidant activity of the extract. However, the best temperature seems to be an intermediate temperature of 65 °C. It is noteworthy that the extraction technique used to obtain the extract with the highest antioxidant activity differed, according to the antioxidant assay used. Regarding the FRAP method, the use of US prior to ST was found to be necessary and was considered the most appropriate extraction. On the contrary, with regards to the DPPH assay, PEF was found to be necessary prior to ST, in order to increase the DPPH free radical scavenging. In a similar study where the extraction parameters were examined for orange peels, the amount of ethanol and the extraction time did not seem to play a significant role, in contrast to temperature where the optimum was 80 °C for the DPPH method. According to the results presented in Table 2, the DPPH values of the extracts range between 10.04 and 616.20 μmol DPPH/g. Therefore, it can be seen that the optimization of the extraction parameters can significantly enhance (up to a 6037% increase) the DPPH scavenging properties of the extracts. Regarding the DPPH method, a suitable extraction technique was the same as in the present study, the combination of PEF and ST. However, time and temperature did not seem to influence the result, which may be due to the different antioxidant compounds contained in the two fruit peels [19].

### 3.5. Ascorbic Acid Content of the Extracts

Among the results presented herein, it is noteworthy that a high amount of ascorbic acid could be extracted. More specifically, using ST with 25% ethanol as the solvent for 150 min at 80 °C, an ascorbic acid quantity of 533.71 ± 86.12 mg/100 g dw (Table 5) could be extracted. According to the results, extraction time and temperature were significant factors in maximizing the extraction yield [47]. In a previous study where quinces were subjected to extraction at a maximum temperature of 50 °C and a time of 10 min, ascorbic acid was recorded at 10 mg/100 g [48,49], while in another study where quinces were extracted at 45 °C to 40 min, the amount of ascorbic acid was found to be only 15.46 mg/100 g [49,50].

### 3.6. Principal Component Analysis (PCA)

**Figure 4:**
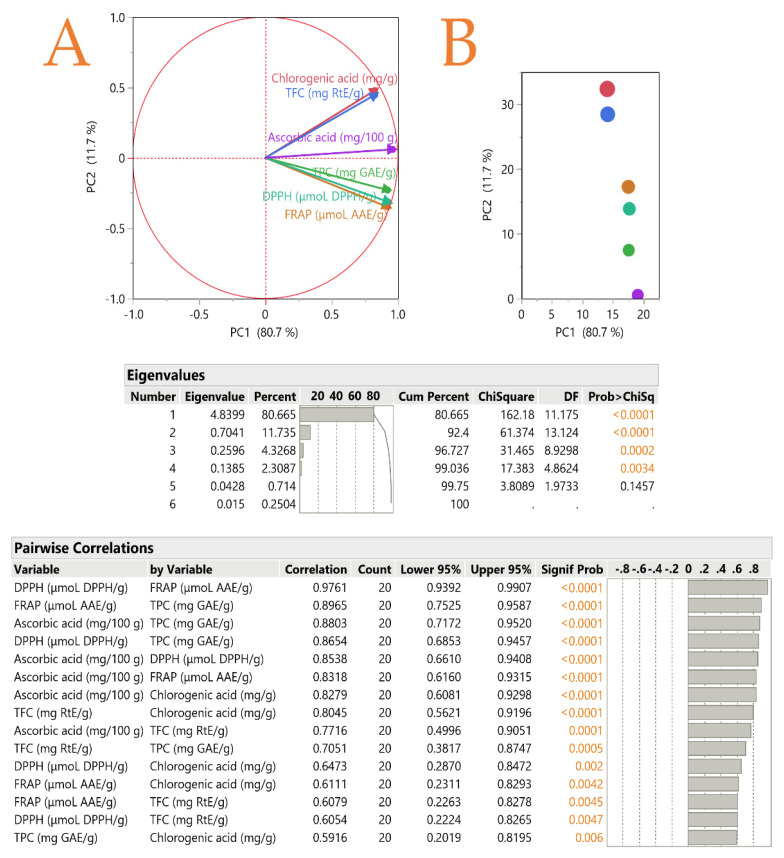
Quince peel extraction plots by various extraction techniques and hydroethanolic solutions, using principal component analysis (PCA). The axis scores for PC1 and PC2 were displayed. Plot (**A**) shows the PCA of the variables and plot (**B**) contains the partial contributions of the variables. One of the six variables used in PCA corresponds to each of the six separate bays, each of which has a different line and color assigned to it. Chlorogenic acid, total polyphenols, total flavonoids, antioxidants (FRAP and DPPH), and ascorbic acid content are examples of physicochemical characteristics. Physicochemical parameters were estimated using pairwise correlation analysis, and colored values indicate statistically significant values.

As can be seen in Figure 4, a critical result is the correlation of similar variables of the samples. For instance, with regards to the methods of evaluating the antioxidant properties of the samples (DPPH and FRAP), the correlation of the values reached 97%, while between different antioxidant compounds such as total polyphenols and ascorbic acid, the correlation of the values was 88%. Furthermore, the correlation between antioxidant capacity and various antioxidants (TPC and ascorbic acid) ranged between 86% to 89%. Such high correlations are not easily achieved and in many cases, it is observed that the correlation among the variables is even lower and sometimes even negative [19].

### 3.7. Partial Least Squares (PLS) Analysis

**Figure 5:**
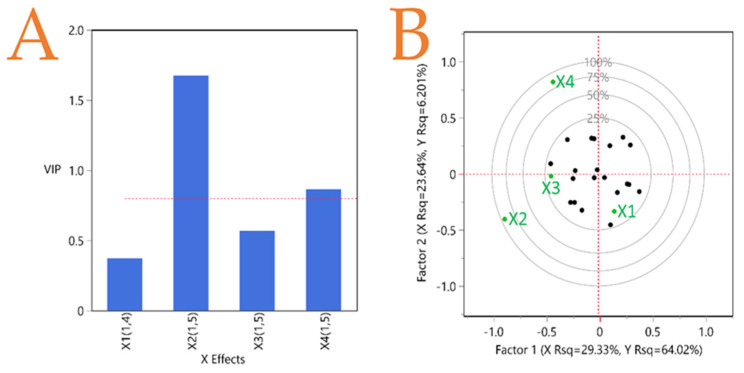
PLS (partial least squares) analysis was used to create graphs showing quince peel extraction using different extraction methods and hydroethanolic solutions. Plot (**A**) shows how many model effects have VIP (variable importance for projection) values greater than 0.8. The correlation loading plot is shown in Plot (**Β**).

A PLS analysis was carried out (Figure 5) to shed light on which of the examined extraction factors (*X*_1_, *X*_2_, *X*_3_, *X*_4_) is the most important. The higher the variable importance for projection (VIP) factor is (when it is greater than 0.8), the more significant the contribution of this factor. According to the results, the most important factor in the extraction of bioactive compounds was found to be *X*_2_ (i.e., solvent concentration), while it was far more important compared to the other examined factors. As mentioned above, the solvent should have a high concentration of ethanol, at least 75%, in order to ensure optimal results for the majority of substances. Factor *X*_4_ (i.e., temperature) also appears to have an influence, but not to a great extent. *X*_3_ and *X*_1_ do not seem to influence the extraction significantly.

**Figure 6:**
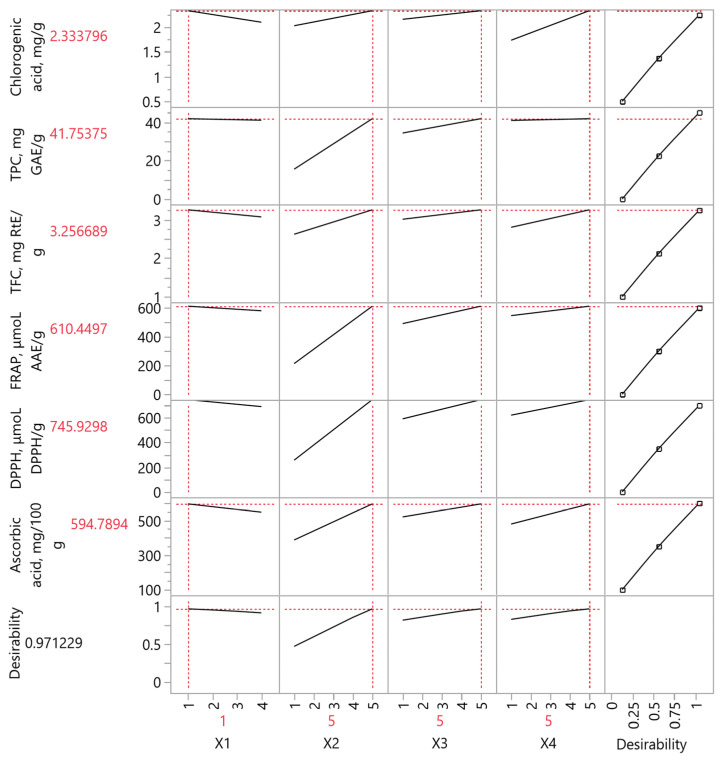
Partial least squares (PLS) prediction profiler of each variable and desirability function with extrapolation control for the optimization of quince peel extracts using different extraction methods and hydroethanolic solutions.

Figure 6 and Table 6 show the ideal extraction conditions for quince peels and the values of the different variables studied throughout the experimental procedure. Upon comparison of the values given by the PLS model with those obtained after experimental analysis, the correlation among them is found to be 0.9976 and they show no deviations with the *p*-value being <0.0001. These results lead to the conclusion that the chemical analyses are quite accurate and are not influenced to a maximum extent by external factors.

## 4. Conclusions

In the present study, various extraction techniques and parameters were studied and optimized in order to obtain the most suitable combination for the extraction of bioactive compounds from waste quince peel. The most appropriate extraction method was found to be ST, a simple and economical method, conducted at a relatively high temperature (65 °C) and for an extended period of time (>120 min). The extraction solvent also seems to play an important role as the higher the percentage of ethanol, the better the extraction. Our results can provide an incentive for further research as antioxidants are extremely important for human health and efforts are constantly being made to produce food products that contain a lot of antioxidants. Finally, it can be concluded that quince peels are a highly promising source of bioactive compounds that if properly utilized, rather than being considered an additional waste good of the food industry, can produce valuable extracts.

## Figures and Tables

**Table 1:** The actual and coded levels of the independent variables were used to optimize the process.

Independent Variables	Code Units	Coded Variable Level				
		1	2	3	4	5
Technique	*X*_1_	ST	PEF + ST	US + ST	PEF + US + ST	–
C (%, *v*/*v*)	*X*_2_	0	25	50	75	100
*t* (min)	*X*_3_	30	60	90	120	150
*T* (°C)	*X*_4_	20	35	50	65	80

**Table 2:** Experimental findings for the four independent variables under investigation and the dependent variable’s responses (Chlorogenic acid, TPC, TFC, FRAP, DPPH, and Ascorbic acid).

Design Point	Independent Variables				Responses					
	*X*_1_	*X*_2_	*X*_3_	*X*_4_	Chlorogenic Acid (mg/g)	TPC (mg GAE/g)	TFC (mg RtE/g)	FRAP (μmol AAE/g)	DPPH (μmol DPPH/g)	Ascorbic Acid (mg/100 g)
1	3	1	3	4	1.63	6.50	2.75	85.68	82.82	214.40
2	3	2	1	3	1.75	21.20	2.69	198.24	179.82	368.46
3	2	3	4	3	1.92	29.32	3.07	318.08	330.14	430.85
4	2	4	5	4	2.27	35.43	3.03	509.33	616.20	527.69
5	3	5	4	2	1.61	38.39	2.96	514.19	567.87	429.83
6	4	1	4	5	1.56	7.58	1.91	132.69	93.44	274.61
7	4	2	3	1	0.59	10.12	1.23	68.76	55.20	158.50
8	1	3	3	2	1.62	28.28	2.54	321.97	301.73	407.73
9	1	4	4	1	1.72	32.78	2.91	434.39	522.21	435.88
10	1	5	1	4	1.79	29.58	2.48	517.90	599.54	434.45
11	1	1	2	3	1.46	8.05	2.10	95.44	74.14	218.30
12	1	2	5	5	2.13	23.25	2.47	331.87	511.45	533.71
13	4	3	2	4	1.83	26.80	2.84	311.36	299.39	418.19
14	3	4	2	5	2.08	35.49	3.05	332.19	371.41	494.74
15	2	5	3	5	1.95	32.33	2.88	489.17	614.33	428.89
16	2	1	1	1	1.29	3.03	1.39	41.47	10.04	114.38
17	2	2	2	2	1.49	20.38	2.34	153.52	89.24	309.33
18	3	3	5	1	1.44	26.10	2.34	165.70	219.08	339.13
19	4	4	1	2	1.77	29.42	2.78	469.83	537.42	440.43
20	4	5	5	3	1.69	37.37	2.17	561.97	584.35	432.84

**Table 3:** Coded values of the four independent variables under investigation and the actual concentration of phenolic compounds, represented in mg/g dw.

Design Point	Independent Variables				Neochlorogenic Acid	Cryptochlorogenic Acid	Rutin	Quercetin 3-*O*-Galactoside	Kaempferol 3-*O*-β-Rutinoside
	X_1_	X_2_	X_3_	X_4_					
1	3	1	3	4	1.35	0.19	0.71	0.11	0.07
2	3	2	1	3	1.29	0.21	0.76	0.12	0.08
3	2	3	4	3	1.34	0.21	0.77	0.12	0.08
4	2	4	5	4	0.95	0.23	0.81	0.13	0.08
5	3	5	4	2	0.67	0.17	0.70	0.11	0.07
6	4	1	4	5	1.16	0.16	0.58	0.09	0.06
7	4	2	3	1	0.54	0.12	0.43	0.05	0.06
8	1	3	3	2	1.18	0.19	0.81	0.14	0.08
9	1	4	4	1	0.68	0.15	0.78	0.13	0.08
10	1	5	1	4	0.70	0.15	0.61	0.11	0.07
11	1	1	2	3	1.12	0.16	0.58	0.08	0.06
12	1	2	5	5	1.51	0.20	0.75	0.13	0.08
13	4	3	2	4	1.32	0.21	0.69	0.11	0.07
14	3	4	2	5	0.90	0.21	0.81	0.13	0.08
15	2	5	3	5	0.75	0.19	0.80	0.13	0.09
16	2	1	1	1	1.09	0.16	0.61	0.09	0.06
17	2	2	2	2	1.05	0.19	0.69	0.10	0.07
18	3	3	5	1	1.08	0.18	0.79	0.13	0.09
19	4	4	1	2	0.83	0.19	0.61	0.10	0.07
20	4	5	5	3	0.68	0.19	0.68	0.11	0.08

**Table 4:** Mathematical models created using RSM were used to optimize the extraction of quince peels from hydroethanolic solutions using various techniques. The models contained only significant terms.

Responses	Second-Order Polynomial Equations (Models)	R^2^	*P*	Equation
Chlorogenic acid	*Y* = 1.05 + 0.18*X*_1_ − 0.11*X*_2_ + 0.06*X*_3_ + 0.13*X*_4_ − 0.07*X*_1_^2^ + 0.02*X*_2_^2^ + 0.08*X*_3_^2^ − 0.06*X*_4_^2^ + 0.04*X*_1_*X*_2_ − 0.11*X*_1_*X*_3_ + 0.11*X*_1_*X*_4_ − 0.05*X*_2_*X*_3_ + 0.03*X*_2_*X*_4_ − 0.01*X*_3_*X*_4_	0.9454	0.0274	(2)
TPC	*Y* = −16.06 + 0.39*X*_1_ + 28.88*X*_2_ − 8.76*X*_3_ + 3.27*X*_4_ − 1.33*X*_1_^2^ − 3.34*X*_2_^2^ − 0.21*X*_3_^2^ + 0.73*X*_4_^2^ + 0.67*X*_1_*X*_2_ + 1.41*X*_1_*X*_3_ − 0.38*X*_1_*X*_4_ + 1.29*X*_2_*X*_3_ − 2.08*X*_2_*X*_4_ + 0.47*X*_3_*X*_4_	0.9806	0.0024	(3)
TFC	*Y* = −1.43 + 1.1*X*_1_ − 0.17*X*_2_ + 1.09*X*_3_ + 0.82*X*_4_ − 0.18*X*_1_^2^ + 0.07*X*_2_^2^ + 0.02*X*_3_^2^ − 0.14*X*_4_^2^ + 0.03*X*_1_*X*_2_ − 0.23*X*_1_*X*_3_ + 0.12*X*_1_*X*_4_ − 0.08*X*_2_*X*_3_ + 0.01*X*_2_*X*_4_ − 0.09*X*_3_*X*_4_	0.9892	0.0006	(4)
FRAP	*Y* = 10.74 − 63.09*X*_1_ + 91.78*X*_2_ − 46.45*X*_3_ + 56*X*_4_ + 16.93*X*_1_^2^ + 7.97*X*_2_^2^ + 9.26*X*_3_^2^ − 15.93*X*_4_^2^ − 3.8*X*_1_*X*_2_ − 16.79*X*_1_*X*_3_ + 8.81*X*_1_*X*_4_ + 0.48*X*_2_*X*_3_ − 4.83*X*_2_*X*_4_ + 14.48*X*_3_*X*_4_	0.9861	0.0011	(5)
DPPH	*Y* = −86.13 + 4.77*X*_1_ − 27.53*X*_2_ + 24.16*X*_3_ + 56.82*X*_4_ + 22.28*X*_1_^2^ + 29.16*X*_2_^2^ + 21.88*X*_3_^2^ − 13.12*X*_4_^2^ − 4.32*X*_1_*X*_2_ − 48.22*X*_1_*X*_3_ + 4.15*X*_1_*X*_4_ − 5.93*X*_2_*X*_3_ + 3.21*X*_2_*X*_4_ + 5.65*X*_3_*X*_4_	0.9816	0.0021	(6)
Ascorbic acid	*Y* = −94.72 − 10.51*X*_1_ + 342.79*X*_2_ − 126.69*X*_3_ + 62.06*X*_4_ − 7.59*X*_1_^2^ − 39.26*X*_2_^2^ + 7.36*X*_3_^2^ + 7.77*X*_4_^2^ + 5.45*X*_1_*X*_2_ + 6.25*X*_1_*X*_3_ − 4.15*X*_1_*X*_4_ + 9.73*X*_2_*X*_3_ − 26.9*X*_2_*X*_4_ + 8.53*X*_3_*X*_4_	0.9776	0.0034	(7)

**Table 5:** Maximum predicted responses and optimum extraction conditions for the dependent variables chlorogenic acid, TPC, TFC, FRAP, DPPH, and ascorbic acid using hydroethanolic solutions.

Responses	Optimal Conditions				
	Maximum Predicted Response	Technique (*X*_1_)	C (%, *v*/*v*) (*X*_2_)	*t* (min) (*X*_3_)	*T* (°C) (*X*_4_)
Chlorogenic acid (mg/g)	2.30 ± 0.41	US + ST (3)	75 (4)	30 (1)	65 (4)
TPC (mg GAE/g)	39.36 ± 7.6	US + ST (3)	100 (5)	150 (5)	35 (2)
TFC (mg RtE/g)	3.49 ± 0.26	US + ST (3)	75 (4)	30 (1)	65 (4)
FRAP (μmol AAE/g)	602.21 ± 98.42	US + ST (3)	100 (5)	150 (5)	65 (4)
DPPH (μmol DPPH/g)	708.06 ± 144.04	PEF + ST (2)	100 (5)	120 (4)	65 (4)
Ascorbic acid (mg/100 g)	533.71 ± 86.12	ST (1)	25 (2)	150 (5)	80 (5)

**Table 6:** Maximum desirability for all variables using the partial least squares (PLS) prediction profiler under the optimal extraction conditions (*X*_1_:1, *X*_2_:5, *X*_3_:5, *X*_4_:5).

Variables	PLS Model Values	Experimental Values
Chlorogenic acid (mg/g)	2.33	2.12
TPC (mg GAE/g)	41.75	43.99
TFC (mg RtE/g)	3.26	3.86
FRAP (μmol AAE/g)	610.45	627.73
DPPH (μmol DPPH/g)	745.93	699.61
Ascorbic acid (mg/100 g)	594.79	543.93

## Data Availability

All related data and methods are presented in this paper. Additional inquiries should be addressed to the corresponding author.

## Supplementary Materials

The following supporting information can be downloaded at: https://www.mdpi.com/article/10.3390/foods12112099/s1, Figure S1. Plots A and B show plots of the actual response versus the predicted response (Chlorogenic acid, mg/g) and the desirability function for the optimization of quince peel extracts performed with hydroethanolic solutions, respectively. Inset tables provide statistics related to the evaluation of the resulting model, and colored values indicate statistically significant values; Figure S2. Plots A and B show plots of the actual response versus the predicted response (Total Polyphenol Content-TPC, mg GAE/g) and the desirability function for the optimization of quince peel extracts performed with hydroethanolic solutions, respectively. Inset tables provide statistics related to the evaluation of the resulting model, and colored values indicate statistically significant values.; Figure S3. Plots A and B show plots of the actual response versus the predicted response (Total Flavonoid Content-TFC, mg RtE/g) and the desirability function for the optimization of quince peel extracts performed with hydroethanolic solutions, respectively. Inset tables provide statistics related to the evaluation of the resulting model, and colored values indicate statistically significant values.; Figure S4. Plots A and B show plots of the actual response versus the predicted response (FRAP, μmol AAE/g) and the desirability function for the optimization of quince peel extracts performed with hydroethanolic solutions, respectively. Inset tables provide statistics related to the evaluation of the resulting model, and colored values indicate statistically significant values.; Figure S5. Plots A and B show plots of the actual response versus the predicted response (DPPH, μmol DPPH/g) and the desirability function for the optimization of quince peel extracts performed with hydroethanolic solutions, respectively. Inset tables provide statistics related to the evaluation of the resulting model, and colored values indicate statistically significant values.; Figure S6. Plots A and B show plots of the actual response versus the predicted response (Ascorbic acid, mg/100 g) and the desirability function for the optimization of quince peel extracts performed with hydroethanolic solutions, respectively. Inset tables provide statistics related to the evaluation of the resulting model, and colored values indicate statistically significant values.; Figure S7. 3D graphs depicting the effect of the process variables considered in the response (Chlorogenic acid, mg/g), to optimize the extraction of the quince peel, using various extraction techniques and hydroethanolic solutions. Plot (A), covariation of *X*_1_ and *X*_2_; plot (B), covariation of *X*_1_ and *X*_3_; plot (C), covariation of *X*_1_ and *X*_4_; plot (D), covariation of *X*_2_ and *X*_3_; plot (E), covariation of *X*_2_ and *X*_4_; plot (F), covariation of *X*_3_ and *X*_4_.; Figure S8. 3D graphs depicting the effect of the process variables considered in the response (Total Flavonoid Content-TFC, mg RtE/g), to optimize the extraction of the quince peel, using various extraction techniques and hydroethanolic solutions. Plot (A), covariation of *X*_1_ and *X*_2_; plot (B), covariation of *X*_1_ and *X*_3_; plot (C), covariation of *X*_1_ and *X*_4_; plot (D), covariation of *X*_2_ and *X*_3_; plot (E), covariation of *X*_2_ and *X*_4_; plot (F), covariation of *X*_3_ and *X*_4_.; Figure S9. 3D graphs depicting the effect of the process variables considered in the response (FRAP, μmol AAE/g), to optimize the extraction of the quince peel, using various extraction techniques and hydroethanolic solutions. Plot (A), covariation of *X*_1_ and *X*_2_; plot (B), covariation of *X*_1_ and *X*_3_; plot (C), covariation of *X*_1_ and *X*_4_; plot (D), covariation of *X*_2_ and *X*_3_; plot (E), covariation of *X*_2_ and *X*_4_; plot (F), covariation of *X*_3_ and *X*_4_.; Figure S10. 3D graphs depicting the effect of the process variables considered in the response (DPPH, μmol DPPH/g), to optimize the extraction of the quince peel, using various extraction techniques and hydroethanolic solutions. Plot (A), covariation of *X*_1_ and *X*_2_; plot (B), covariation of *X*_1_ and *X*_3_; plot (C), covariation of *X*_1_ and *X*_4_; plot (D), covariation of *X*_2_ and *X*_3_; plot (E), covariation of *X*_2_ and *X*_4_; plot (F), covariation of *X*_3_ and *X*_4_.
