# Supplementary material for: Optimization of Extraction Parameters for Enhanced Recovery of Bioactive Compounds from Quince Peels Using Response Surface Methodology
Source: Foods. 2023 May 23;12(11):2099. doi: 10.3390/foods12112099 (PMC10252653; doi:10.3390/foods12112099)
Supplement: Supplementary file 1 [file foods-12-02099-s001.zip › foods-2397727-supplementary.pdf]

Supplementary material

Optimization of Extraction Parameters for Enhanced Recovery of Bioactive Compounds from Quince Peels Using Response Surface Methodology

Vassilis Athanasiadis, Theodoros Chatzimitakos, Eleni Bozinou, Konstantina Kotsou, Dimitrios Palaiogiannis and Stavros I. Lalas *

Department of Food Science & Nutrition, University of Thessaly, Terma N. Temponera Str., 43100 Karditsa, Greece

* Correspondence: slalas@uth.gr; Tel.: +30-24410-64783

2.1 Chemicals and reagents

All of the solvents were purchased from Carlo Erba in Val de Reuil, France, and were at least of HPLC grade. Penta (Prague, Czech Republic) provided the gallic acid, anhydrous sodium carbonate, 2,4,6-tri-2-pyridinyl-1,3,5-triazine (TPTZ), and Folin-Ciocalteu reagent. Chemical standards for the HPLC-based determination of polyphenols [i.e., chlorogenic acid (3-*O*-caffeoylquinic acid), neochlorogenic acid (5-*O*-caffeoylquinic acid), cryptochlorogenic acid (4-*O*-caffeoylquinic acid), rutin, quercetin 3-*O*-galactoside, and kaempferol 3-*O*-β-rutinoside], aluminum chloride, iron (III) chloride, hydrochloric acid, ascorbic acid, and trichloroacetic acid were purchased from Sigma-Aldrich (Steinheim, Germany). Deionized water was used for all conducted experiments.

2.4. Determination of total polyphenol content (TPC)

Briefly, 100 μL of quince peel extracts were mixed with 100 μL of Folin-Ciocalteu reagent in a 1.5 mL Eppendorf tube. After 2 min, 800 μL of Na_2CO_3 solution (5% *w*/*v*) was added and the solutions were incubated at 40 °C for 20 min. Eventually, the absorbance at 740 nm was determined using a Shimadzu spectrophotometer (UV-1700, Shimadzu Europa GmbH, Duisburg, Germany). Gallic acid was used to create a calibration curve (10–80 mg/L) using a standard compound. The TPC (C_{TP}) was expressed as mg gallic acid equivalents (GAE) per L. Equation (S1) was used to express the extraction yield in terms of total polyphenols (Y_{TP}) as mg GAE per g of dry weight (dw):

$$Y_{TP}\ (\text{mg GAE/g dw}) = \frac{C_{TP} \times V}{w} \quad \text{(S1)}$$

where V is the volume of the extraction medium (in L) and w is the dry weight of the sample (in g).

2.5. Determination of total flavonoid content (TFC)

100 μL of the extract was mixed with 860 μL of aqueous ethanol (35% *v*/*v*) and 0.04 μL of a reagent containing 0.5 M sodium acetate and 5% *w*/*v* aluminum chloride in an Eppendorf tube. The resulting mixture was vortexed and reacted at room temperature for 30 minutes. The absorbance at 415 nm was then measured against a suitable blank. The concentration of total flavonoids (TFC), expressed in mg/L, was estimated by calibration curve (20–100 mg/L), which was constructed by plotting known rutin solutions against absorbance at 415 nm. The TFC was expressed as mg rutin equivalents (RtE) per g dw, using the following Equation (S2):

$$\text{TFC (mg RtE/g dw)} = \frac{C_{TFc} \times V}{w} \quad \text{(S2)}$$

where, V is the volume of the extraction medium (in L), and w is the dry weight of the sample (in g).

2.6. Ferric reducing antioxidant power (FRAP) assay

$FeCl_3$ solution (4 mM in 0.05 M HCl) was combined with 0.05 mL of the extract in an Eppendorf tube, and the mixture was then incubated at 37 °C for 30 min. Following the addition of 0.90 mL of the TPTZ solution (1 mM in 0.05 M HCl), the absorbance at 620 nm was measured after 5 minutes. Ascorbic acid was used as a standard compound to produce a calibration curve (C_{AA}, 50–500 moL/L in 0.05 M HCl). Equation (S3) was used to calculate the ferric reducing antioxidant power (P_R) as μmoL ascorbic acid equivalents (AAE) per g of dw:

$$P_R \text{ (μmoL AAE/g dw)} = \frac{C_{AA} \times V}{w} \qquad \text{(S3)}$$

where V is the volume of the extraction medium (in L) and w is the dry weight of the sample (in g).

2.7. DPPH Radical Scavenging Activity

Briefly, 25 μL of the prepared extract was added to 975 μL of 100 μM DPPH solution, thoroughly mixed, and the solution's absorbance was measured at 515 nm ($A_{515(i)}$) and after 30 min of incubation in the dark ($A_{515(f)}$). Equation (S4) was utilized to compute the antiradical activity (A_{AR}):

$$A_{AR} \text{ (μmoL DPPH/g dw)} = \frac{\Delta A}{\varepsilon \times l \times C} \times Y_{TP} \qquad \text{(S4)}$$

where $\Delta A = A_{515(i)} - A_{515(f)}$; ε (DPPH) = 11,126 × 10^{-6} μM^{-1} cm^{-1}; $C = C_{TP} \times 0.025$; Y_{TP} is the total polyphenol yield of the extract (mg/g), and l is the path length (1 cm).

2.9. HPLC-based determination of the chlorogenic acid content and other phenolic compounds

The analysis was conducted using a Shimadzu CBM-20A liquid chromatograph and a Shimadzu SPD-M20A diode array detector (both supplied by Shimadzu Europa GmbH, Duisburg, Germany). The compounds were separated using a Phenomenex Luna C18(2) column from Phenomenex Inc. in Torrance, California, maintained at 40 °C (100 Å, 5 μm, 4.6 × 250 mm). The mobile phase consisted of 0.5% aqueous formic acid (A) and a mixture of 0.5% formic acid in acetonitrile/water (6:4) (B). The gradient program used was as follows: 0% B to 40% B, then to 50% B in 10 min, to 70% B in another 10 min and then held constant for 10 min. The flow rate of the mobile phase was 1 mL/min. The retention time and absorbance spectrum were compared to those of pure chemical standards to identify the compounds and then quantified using calibration curves (0–50 μg/mL).

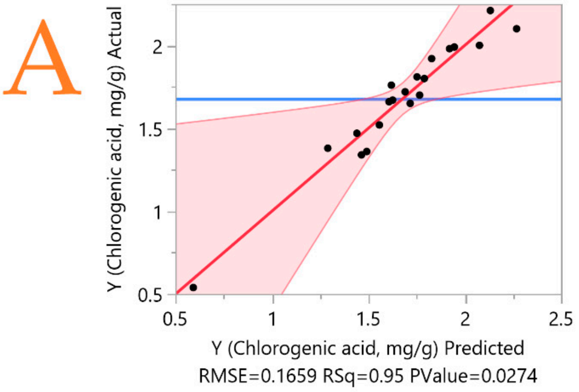

Analysis of Variance

Source	DF	Sum of Squares	Mean Square	F Ratio
Model	14	2.3841	0.1703	6.1841
Error	5	0.1377	0.0275	**Prob > F**
C. Total	19	2.5218		0.0274

Summary of Fit

RSquare	0.9454
RSquare Adj	0.7925
Root Mean Square Error	0.1659
Mean of Response	1.679
Observations (or Sum Wgts)	20

Parameter Estimates

Term	Estimate	Std Error	t Ratio	Prob>|t|
Intercept	1.7032	0.1376	12.376	0.0001
X1(1,4)	-0.108	0.0588	-1.829	0.127
X2(1,5)	0.1299	0.0981	1.324	0.2428
X3(1,5)	0.1423	0.1419	1.0022	0.3623
X4(1,5)	0.2515	0.0927	2.7121	0.0422
X1*X1	-0.167	0.1049	-1.595	0.1717
X1*X2	0.1178	0.103	1.1429	0.3048
X2*X2	0.0893	0.2385	0.3745	0.7234
X1*X3	-0.316	0.1929	-1.639	0.1622
X2*X3	-0.206	0.2195	-0.94	0.3902
X3*X3	0.306	0.1168	2.6206	0.0471
X1*X4	0.318	0.1435	2.216	0.0775
X2*X4	0.1286	0.2352	0.5465	0.6082
X3*X4	-0.035	0.1351	-0.262	0.8041
X4*X4	-0.229	0.1765	-1.295	0.2517

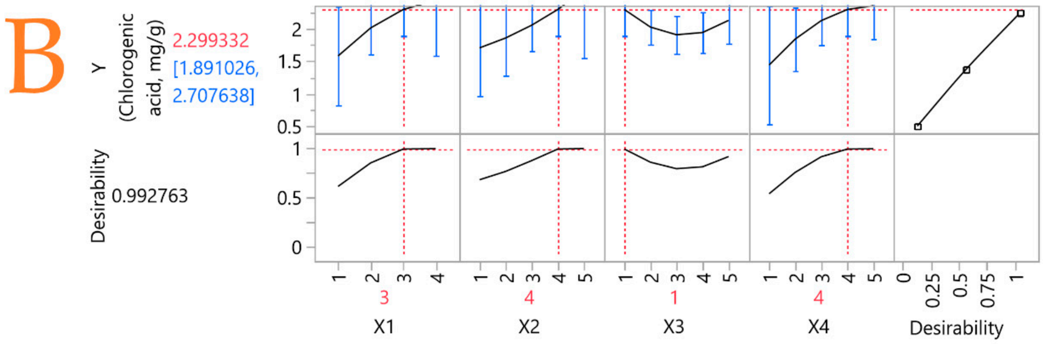

Figure S1. Plots A and B show plots of the actual response versus the predicted response (Chlorogenic acid, mg/g) and the desirability function for the optimization of quince peel extracts performed with hydroethanolic solutions, respectively. Inset tables provide statistics related to the evaluation of the resulting model, and colored values indicate statistically significant values.

A

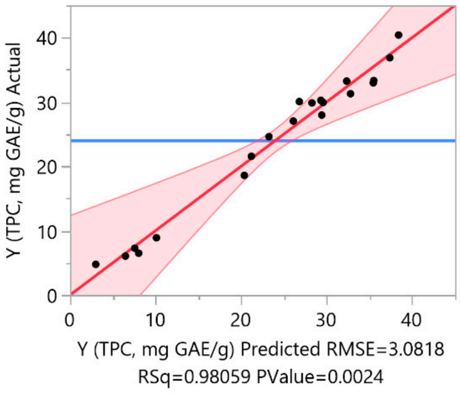

Analysis of Variance

Source	DF	Sum of Squares	Mean Square	F Ratio
Model	14	2398.7	171.33	18.04
Error	5	47.486	9.4972	**Prob > F**
C. Total	19	2446.2		0.0024

Parameter Estimates

Term	Estimate	Std Error	t Ratio	Prob>|t|
Intercept	31.172	2.5557	12.197	0.0001
X1(1,4)	-1.8	1.0918	-1.648	0.1602
X2(1,5)	16.246	1.8216	8.9185	0.0003
X3(1,5)	-2.424	2.6361	-0.92	0.4
X4(1,5)	3.7017	1.7221	2.1495	0.0843
X1*X1	-2.994	1.9477	-1.537	0.1849
X1*X2	1.9952	1.9135	1.0427	0.3449
X2*X2	-13.37	4.4289	-3.019	0.0294
X1*X3	4.2201	3.583	1.1778	0.2919
X2*X3	5.1526	4.0771	1.2638	0.262
X3*X3	-0.831	2.1682	-0.383	0.7172
X1*X4	-1.154	2.665	-0.433	0.683
X2*X4	-8.307	4.3685	-1.902	0.1156
X3*X4	1.8812	2.5093	0.7497	0.4872
X4*X4	2.9066	3.2778	0.8867	0.4158

Summary of Fit

RSquare	0.9806
RSquare Adj	0.9262
Root Mean Square Error	3.0818
Mean of Response	24.071
Observations (or Sum Wgts)	20

B

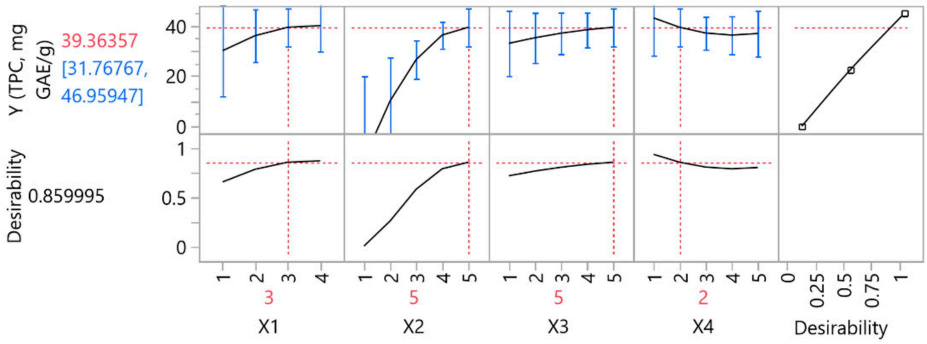

Figure S2. Plots A and B show plots of the actual response versus the predicted response (Total Polyphenol Content-TPC, mg GAE/g) and the desirability function for the optimization of quince peel extracts performed with hydroethanolic solutions, respectively. Inset tables provide statistics related to the evaluation of the resulting model, and colored values indicate statistically significant values.

A

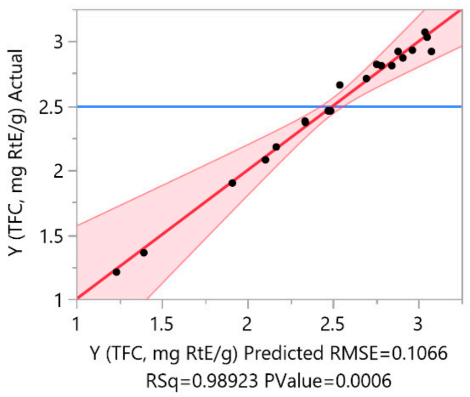

Analysis of Variance

Source	DF	Sum of Squares	Mean Square	F Ratio
Model	14	5.2247	0.3732	32.813
Error	5	0.0569	0.0114	**Prob > F**
C. Total	19	5.2816		0.0006

Parameter Estimates

Term	Estimate	Std Error	t Ratio	Prob>|t|
Intercept	2.8711	0.0884	32.462	0.0001
X1(1,4)	-0.104	0.0378	-2.742	0.0407
X2(1,5)	0.2486	0.063	3.9436	0.0109
X3(1,5)	0.161	0.0912	1.7643	0.1379
X4(1,5)	0.1335	0.0596	2.2395	0.0753
X1*X1	-0.414	0.0674	-6.136	0.0017
X1*X2	0.0907	0.0662	1.37	0.229
X2*X2	0.2814	0.1533	1.8363	0.1257
X1*X3	-0.703	0.124	-5.668	0.0024
X2*X3	-0.328	0.1411	-2.327	0.0675
X3*X3	0.0628	0.075	0.8364	0.441
X1*X4	0.3638	0.0922	3.9447	0.0109
X2*X4	0.0584	0.1512	0.3861	0.7153
X3*X4	-0.362	0.0868	-4.165	0.0088
X4*X4	-0.552	0.1134	-4.863	0.0046

Summary of Fit

RSquare	0.9892
RSquare Adj	0.9591
Root Mean Square Error	0.1066
Mean of Response	2.4975
Observations (or Sum Wgts)	20

B

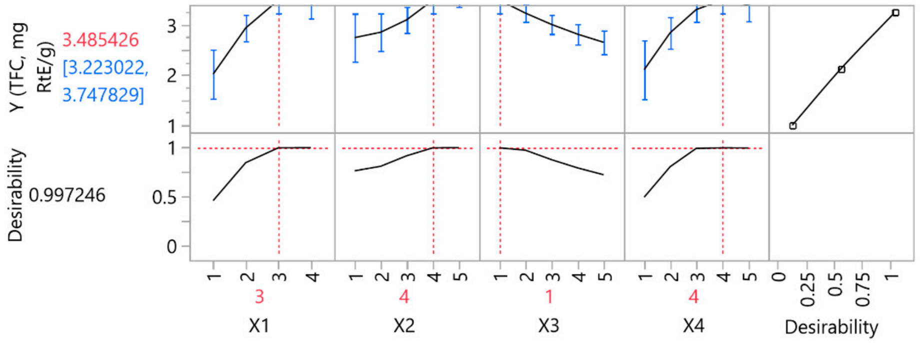

Figure S3. Plots A and B show plots of the actual response versus the predicted response (Total Flavonoid Content-TFC, mg RtE/g) and the desirability function for the optimization of quince peel extracts performed with hydroethanolic solutions, respectively. Inset tables provide statistics related to the evaluation of the resulting model, and colored values indicate statistically significant values.

A

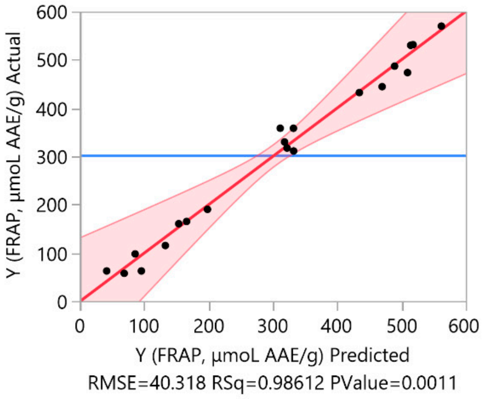

Analysis of Variance

Source	DF	Sum of Squares	Mean Square	F Ratio
Model	14	577266	41,233	25.366
Error	5	8127.8	1625.6	**Prob > F**
C. Total	19	585393		0.0011

Parameter Estimates

Term	Estimate	Std Error	t Ratio	Prob>|t|
Intercept	277.31	33.436	8.2935	0.0004
X1(1,4)	-20.66	14.284	-1.447	0.2076
X2(1,5)	234.16	23.832	9.8255	0.0002
X3(1,5)	23.998	34.488	0.6958	0.5175
X4(1,5)	22.743	22.53	1.0094	0.3591
X1*X1	38.095	25.481	1.495	0.1951
X1*X2	-11.39	25.034	-0.455	0.6681
X2*X2	31.899	57.943	0.5505	0.6057
X1*X3	-50.38	46.876	-1.075	0.3316
X2*X3	1.9037	53.34	0.0357	0.9729
X3*X3	37.048	28.366	1.306	0.2484
X1*X4	26.431	34.866	0.7581	0.4826
X2*X4	-19.3	57.153	-0.338	0.7493
X3*X4	57.914	32.829	1.7641	0.138
X4*X4	-63.74	42.883	-1.486	0.1973

Summary of Fit

RSquare	0.9861
RSquare Adj	0.9472
Root Mean Square Error	40.318
Mean of Response	302.69
Observations (or Sum Wgts)	20

B

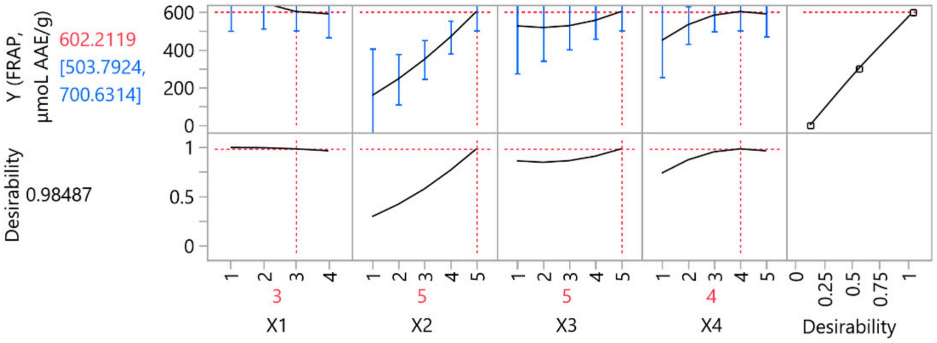

Figure S4. Plots A and B show plots of the actual response versus the predicted response (FRAP, µmoL AAE/g) and the desirability function for the optimization of quince peel extracts performed with hydroethanolic solutions, respectively. Inset tables provide statistics related to the evaluation of the resulting model, and colored values indicate statistically significant values.

A

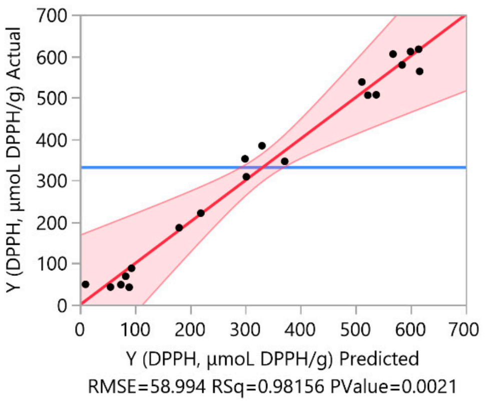

Analysis of Variance

Source	DF	Sum of Squares	Mean Square	F Ratio
Model	14	926083	66,149	19.007
Error	5	17,401	3480.2	**Prob > F**
C. Total	19	943484		0.0021

Parameter Estimates

Term	Estimate	Std Error	t Ratio	Prob>|t|
Intercept	230.05	48.924	4.7022	0.0053
X1(1,4)	-43.52	20.901	-2.082	0.0918
X2(1,5)	256.87	34.871	7.3663	0.0007
X3(1,5)	68.052	50.463	1.3486	0.2353
X4(1,5)	30.161	32.966	0.9149	0.4022
X1*X1	50.129	37.284	1.3445	0.2366
X1*X2	-12.97	36.63	-0.354	0.7377
X2*X2	116.63	84.782	1.3756	0.2274
X1*X3	-144.7	68.589	-2.109	0.0887
X2*X3	-23.73	78.047	-0.304	0.7733
X3*X3	87.515	41.505	2.1085	0.0888
X1*X4	12.464	51.015	0.2443	0.8167
X2*X4	12.838	83.626	0.1535	0.884
X3*X4	22.596	48.035	0.4704	0.6579
X4*X4	-52.47	62.747	-0.836	0.4412

Summary of Fit

RSquare	0.9816
RSquare Adj	0.9299
Root Mean Square Error	58.994
Mean of Response	332.99
Observations (or Sum Wgts)	20

B

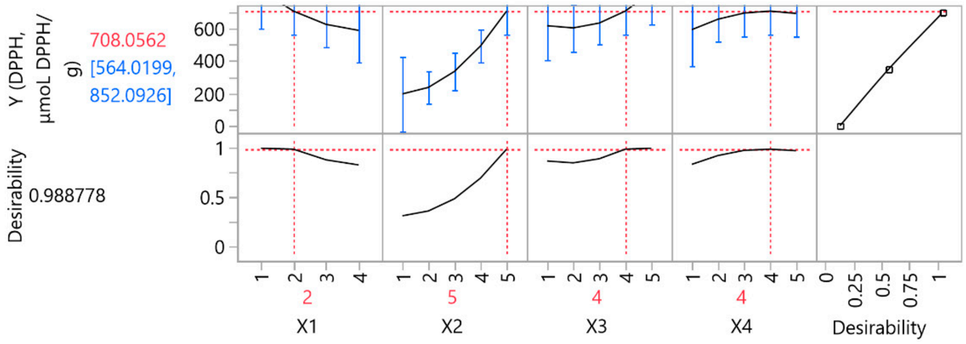

Figure S5. Plots A and B show plots of the actual response versus the predicted response (DPPH, µmoL DPPH/g) and the desirability function for the optimization of quince peel extracts performed with hydroethanolic solutions, respectively. Inset tables provide statistics related to the evaluation of the resulting model, and colored values indicate statistically significant values.

A

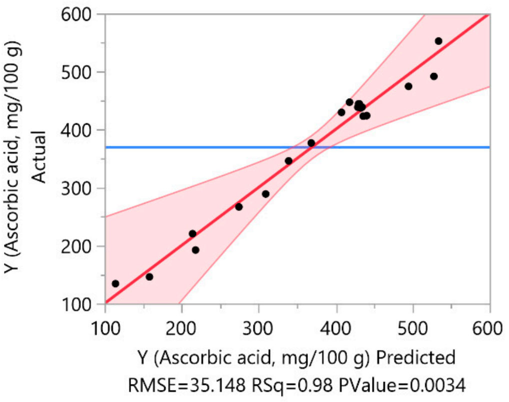

Analysis of Variance

Source	DF	Sum of Squares	Mean Square	F Ratio
Model	14	270155	19,297	15.62
Error	5	6177.1	1235.4	**Prob > F**
C. Total	19	276332		0.0034

Summary of Fit

RSquare	0.9776
RSquare Adj	0.9151
Root Mean Square Error	35.148
Mean of Response	370.62
Observations (or Sum Wgts)	20

Parameter Estimates

Term	Estimate	Std Error	t Ratio	Prob>|t|
Intercept	427.73	29.149	14.674	0.0001
X1(1,4)	-38.72	12.453	-3.109	0.0266
X2(1,5)	138.71	20.776	6.6762	0.0011
X3(1,5)	-24.23	30.066	-0.806	0.4569
X4(1,5)	86.34	19.641	4.3959	0.007
X1*X1	-17.09	22.214	-0.769	0.4765
X1*X2	16.363	21.824	0.7498	0.4871
X2*X2	-157	50.513	-3.109	0.0266
X1*X3	18.761	40.866	0.4591	0.6654
X2*X3	38.924	46.5	0.8371	0.4407
X3*X3	29.435	24.729	1.1903	0.2874
X1*X4	-12.45	30.395	-0.41	0.699
X2*X4	-107.6	49.824	-2.16	0.0832
X3*X4	34.133	28.619	1.1927	0.2865
X4*X4	31.066	37.385	0.831	0.4438

B

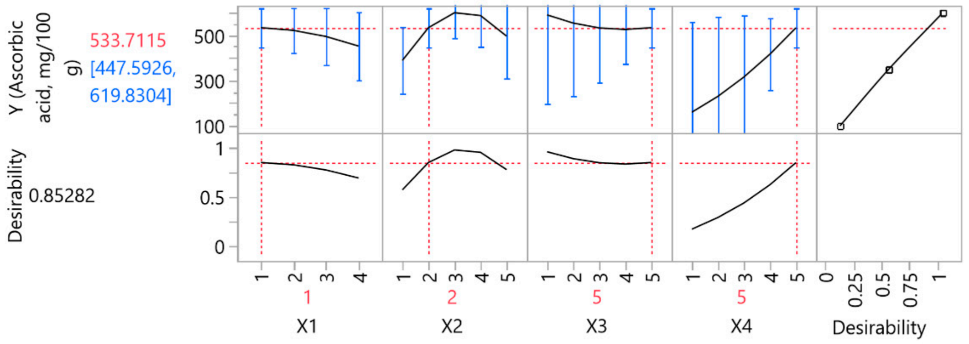

Figure S6. Plots A and B show plots of the actual response versus the predicted response (Ascorbic acid, mg/100 g) and the desirability function for the optimization of quince peel extracts performed with hydroethanolic solutions, respectively. Inset tables provide statistics related to the evaluation of the resulting model, and colored values indicate statistically significant values.

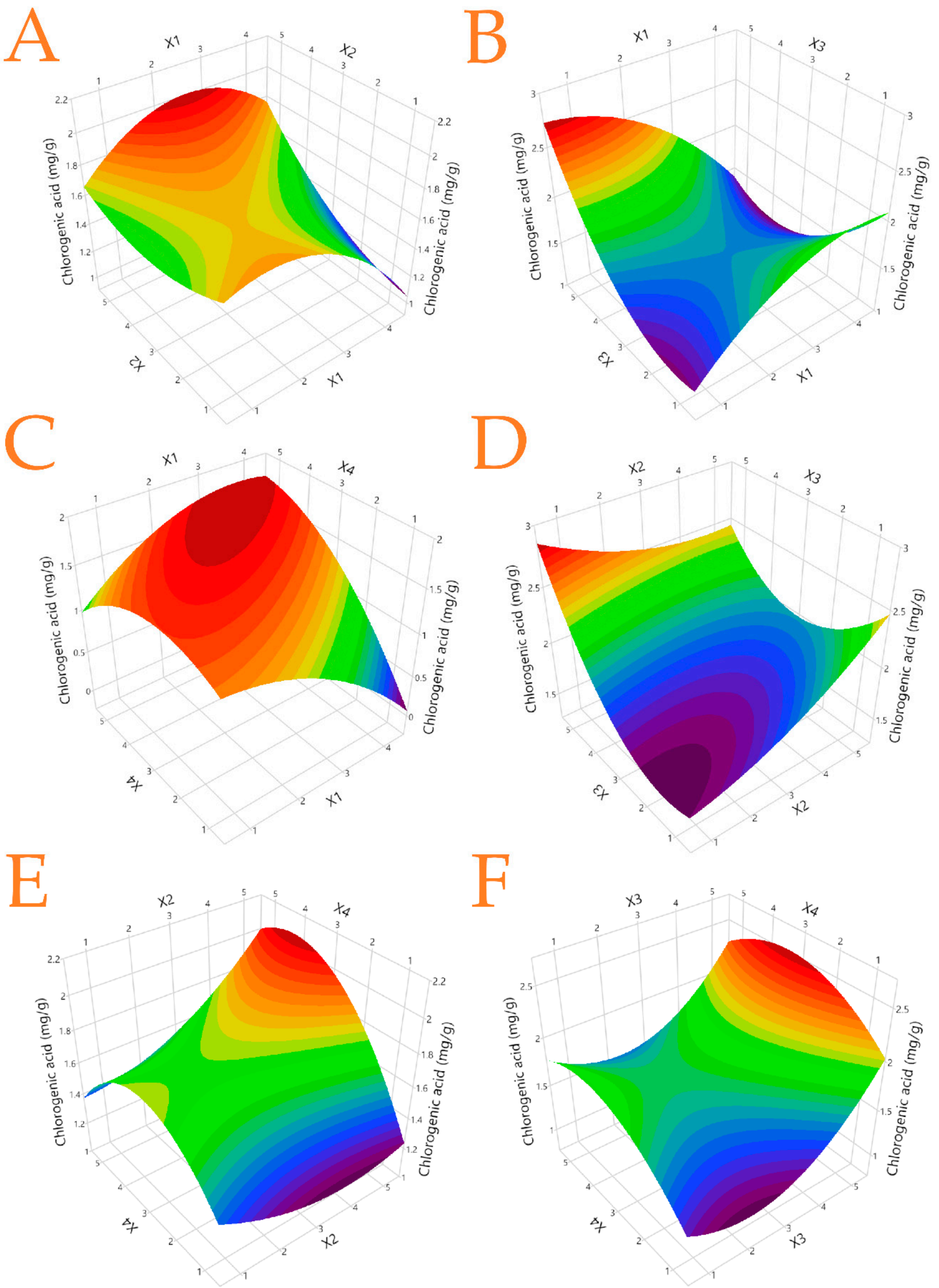

Figure S7. 3D graphs depicting the effect of the process variables considered in the response (Chlorogenic acid, mg/g), to optimize the extraction of the quince peel, using various extraction techniques and hydroethanolic solutions. Plot (A), covariation of X_1 and X_2; plot (B), covariation of X_1 and X_3; plot (C), covariation of X_1 and X_4; plot (D), covariation of X_2 and X_3; plot (E), covariation of X_2 and X_4; plot (F), covariation of X_3 and X_4.

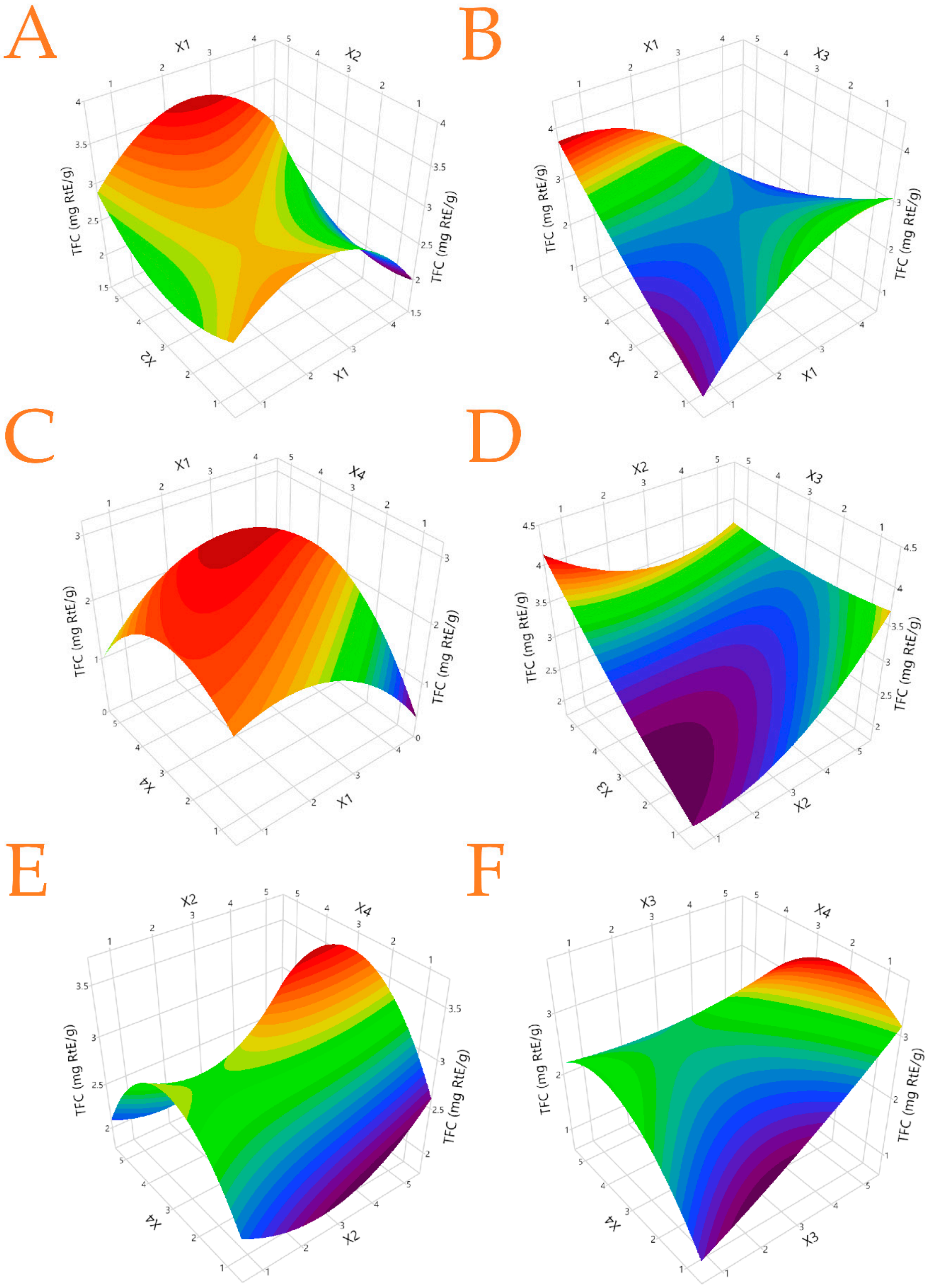

Figure S8. 3D graphs depicting the effect of the process variables considered in the response (Total Flavonoid Content-TFC, mg RtE/g), to optimize the extraction of the quince peel, using various extraction techniques and hydroethanolic solutions. Plot (A), covariation of X_1 and X_2; plot (B), covariation of X_1 and X_3; plot (C), covariation of X_1 and X_4; plot (D), covariation of X_2 and X_3; plot (E), covariation of X_2 and X_4; plot (F), covariation of X_3 and X_4.

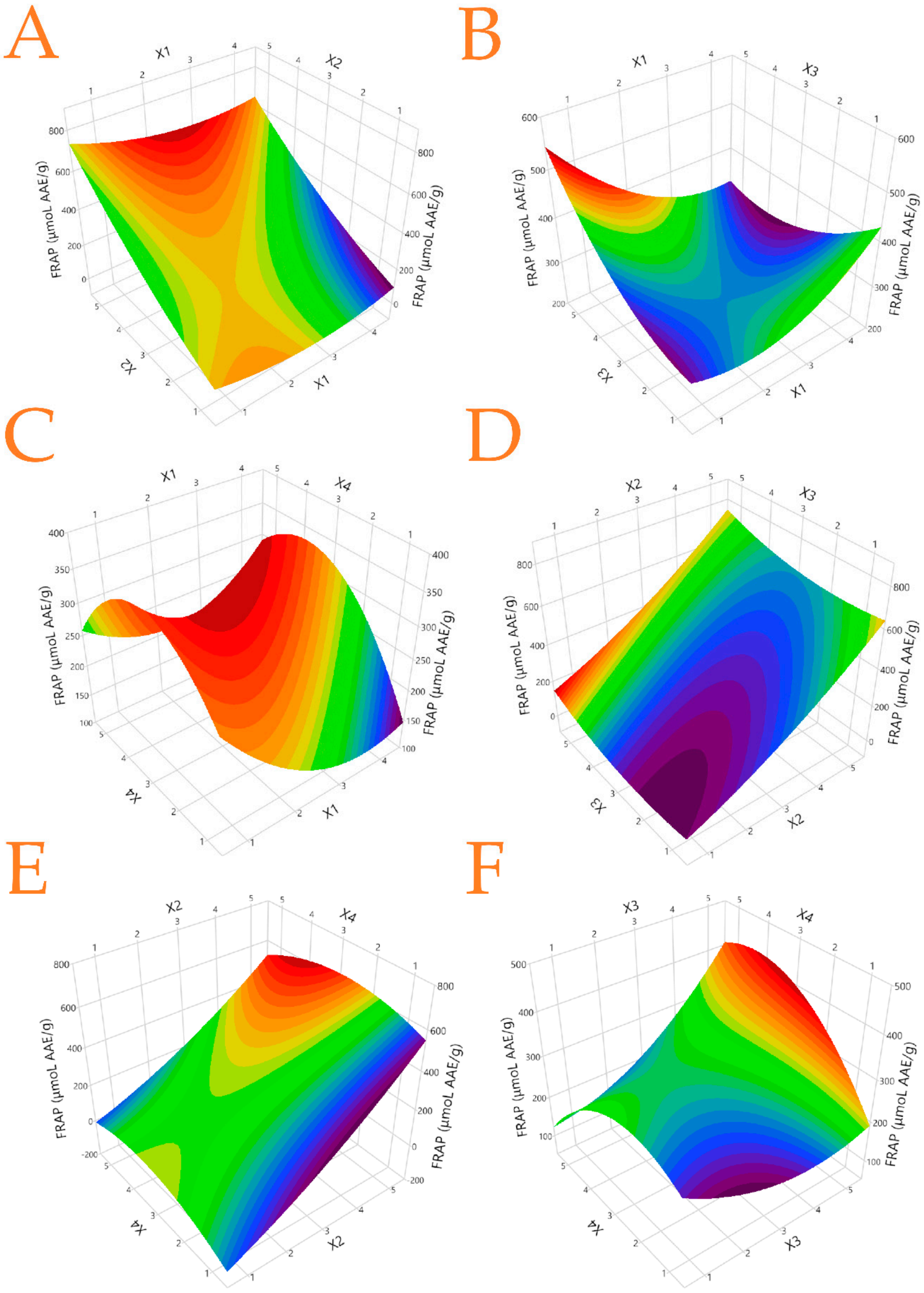

Figure S9. 3D graphs depicting the effect of the process variables considered in the response (FRAP, µmoL AAE/g), to optimize the extraction of the quince peel, using various extraction techniques and hydroethanolic solutions. Plot (A), covariation of X_1 and X_2; plot (B), covariation of X_1 and X_3; plot (C), covariation of X_1 and X_4; plot (D), covariation of X_2 and X_3; plot (E), covariation of X_2 and X_4; plot (F), covariation of X_3 and X_4.

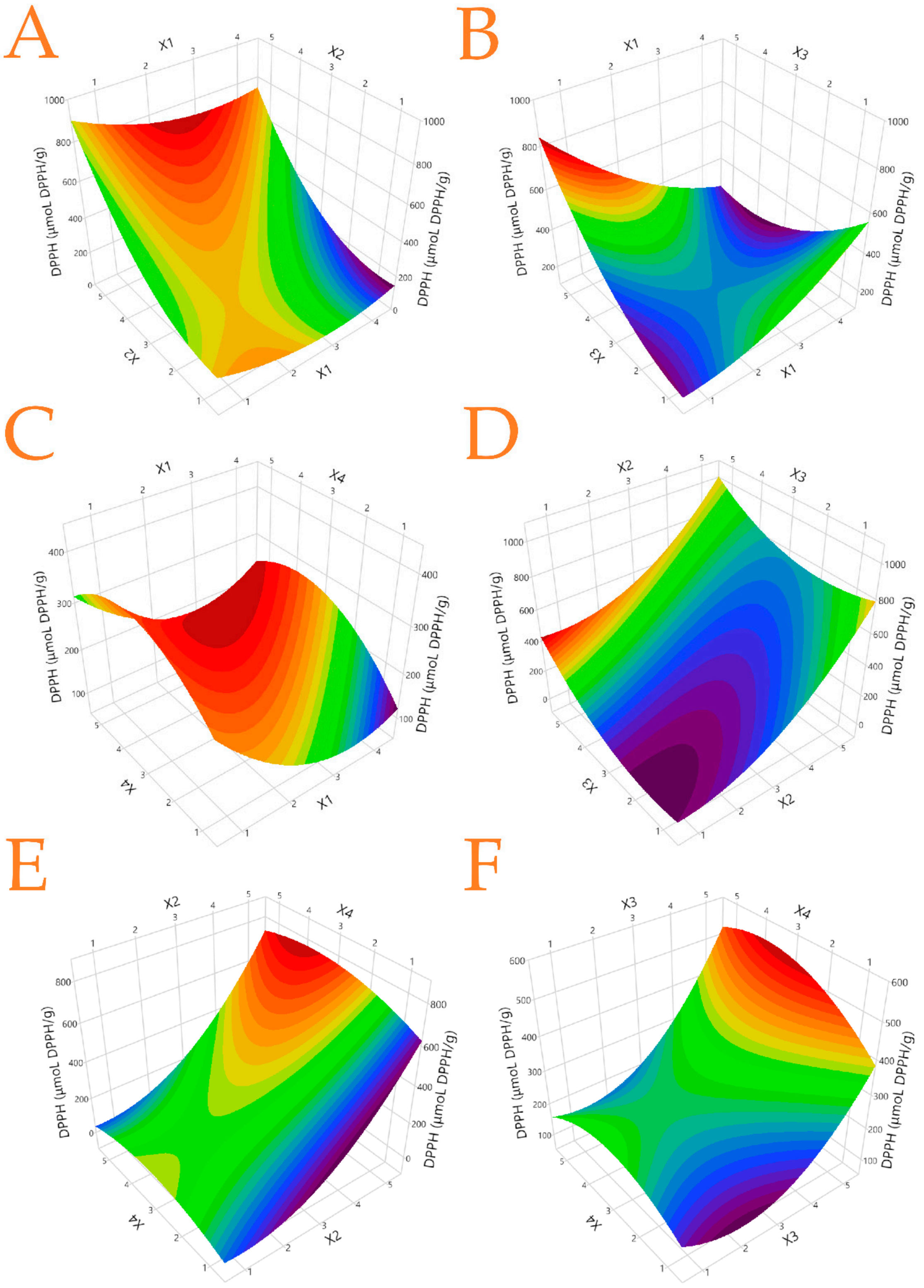

Figure S10. 3D graphs depicting the effect of the process variables considered in the response (DPPH, µmoL DPPH/g), to optimize the extraction of the quince peel, using various extraction techniques and hydroethanolic solutions. Plot (A), covariation of X_1 and X_2; plot (B), covariation of X_1 and X_3; plot (C), covariation of X_1 and X_4; plot (D), covariation of X_2 and X_3; plot (E), covariation of X_2 and X_4; plot (F), covariation of X_3 and X_4.
